# Clinical efficacy and tumour microenvironment influence of decitabine plus R‐CHOP in patients with newly diagnosed diffuse large B‐Cell lymphoma: Phase 1/2 and biomarker study

**DOI:** 10.1002/ctm2.584

**Published:** 2021-12-19

**Authors:** Mu‐Chen Zhang, Ying Fang, Peng‐Peng Xu, Lei Dong, Rong Shen, Yao‐Hui Huang, Di Fu, Zi‐Xun Yan, Shu Cheng, Xu‐Feng Jiang, Qi Song, Yang He, Yan Zhao, Min Lu, Jing Ye, Feng Liu, Lin Cheng, Chao‐Fu Wang, Li Wang, Wei‐Li Zhao

**Affiliations:** ^1^ Shanghai Institute of Hematology, State Key Laboratory of Medical Genomics, National Research Center for Translational Medicine at Shanghai Ruijin Hospital affiliated to Shanghai Jiao Tong University School of Medicine Shanghai China; ^2^ Department of Hematology Shanghai Jiao Tong University Affiliated Sixth People's Hospital Shanghai China; ^3^ Department of Pathology Ruijin Hospital affiliated to Shanghai Jiao Tong University School of Medicine Shanghai China; ^4^ Department of Nuclear Medicine Ruijin Hospital affiliated to Shanghai Jiao Tong University School of Medicine Shanghai China; ^5^ Department of Radiology Ruijin Hospital affiliated to Shanghai Jiao Tong University School of Medicine Shanghai China; ^6^ Laboratory of Molecular Pathology Pôle de Recherches Sino‐Français en Science du Vivant et Génomique Shanghai China

Dear Editor,

Epigenetic gene alterations play an important role on diffuse large B cell lymphoma (DLBCL) progression.^1^ DNA methyltransferase inhibitor (DNMTi) decitabine has demonstrated anti‐lymphoma activities, but never been applied for newly diagnosed DLBCL treatment. Here, we conducted for the first time a phase 1/2 trial of decitabine plus standard immunochemotherapy rituximab, cyclophosphamide, doxorubicin, vincristine and prednisone (DR‐CHOP, NCT02951728) in DLBCL patients with international prognostic index (IPI) ≥ 2. The study determined the maximum tolerated dose (MTD) of decitabine as 10 mg/m^2^ on days 1–5 prior to R‐CHOP on days 6–11 and showed promising efficacy and good tolerability.

The trial enrolled 54 patients, 11 in phase 1 and 43 in phase 2 (Table 1). Among 49 evaluable patients (including six patients received the MTD of decitabine in phase 1), 39 (79.6%) patients achieved complete remission, and six (12.2%) patients achieved partial remission. Two‐year progression‐free survival (PFS), event‐free survival (EFS) and overall survival (OS) rates were 71.4%, 65.3% and 87.8%, respectively (Figure 1A). Intermediate‐high (IPI 2–3) or high‐risk (IPI 4–5) patients presented similar outcomes (Figure 1B), irrespective on cell of origin and BCL2/MYC double expression (as defined by immunohistochemistry BCL2 ≥ 50% and MYC ≥ 40%) (Figure S1A,B). In our previous cohort of NHL‐001 (NCT01852435), 2‐year PFS was 59.6%, with OS as 76.2% for IPI ≥ 2 patients with standard R‐CHOP (R‐CHOP50 and R‐CEOP70)^2^ (Figure 1C). The main adverse events (Table S1) were grade 3–4 hematological toxicity, particularly grade 3–4 neutropenia, comparable to other novel targeted agents plus R‐CHOP as ibrutinib, lenalidomide and venetoclax^3^ and manageable with granulocyte‐colony stimulating factor prophylaxis and supportive care.

**TABLE 1:** Baseline characteristics of the enrolled patients

	Phase 1 (*n* = 11)	Phase 2 (*n* = 43)	Evaluable* (*n* = 49)
Age: median (range)	46 (25–57)	56 (29–74)	55 (25–74)
≤60	11 (100%)	25 (58.1%)	31 (63.3%)
>60	0	18 (41.9%)	18 (36.7%)
Gender			
Male	3 (27.3%)	20 (46.5%)	22 (44.9%)
Female	8 (72.7%)	23 (53.5%)	27 (55.1%)
ECOG			
0–1	11 (100%)	34 (79.1%)	40 (81.6%)
2	0	9 (20.9%)	9 (18.4%)
Ann Arbor stage			
II	1 (9.1%)	4 (9.3%)	5 (10.2%)
III–IV	10 (90.9%)	39 (90.7%)	44 (89.8%)
LDH			
Normal	2 (18.2%)	5 (11.6%)	6 (12.2%)
Elevated	9 (81.8%)	38 (88.4%)	43 (87.8%)
Extranodal sites			
0–1	2 (18.2%)	12 (27.9%)	12 (24.5%)
≥2	9 (81.8%)	31 (72.1%)	37 (75.5%)
IPI			
2	5 (45.5%)	11 (25.6%)	14 (28.6%)
3	6 (54.5%)	17 (39.5%)	20 (40.8%)
4‐5	0	15 (34.9%)	15 (30.6%)
Cell of origin			
GCB	3 (27.3%)	16 (37.2%)	19 (38.8%)
Non‐GCB	8 (72.7%)	27 (62.8%)	30 (61.2%)
BCL2/MYC double expression			
Yes	3 (27.3%)	5 (11.6%)	7 (14.3%)
No	8 (72.7%)	38 (88.4%)	42 (85.7%)

Abbreviations: ECOG, Eastern Cooperative Oncology Group. LDH, lactate dehydrogenase. IPI, international prognostic index. GCB, germinal center B‐cell.

* Evaluable patients included 6 patients from phase 1 who received 10 mg/m^2^ decitabine.

**FIGURE 1:**
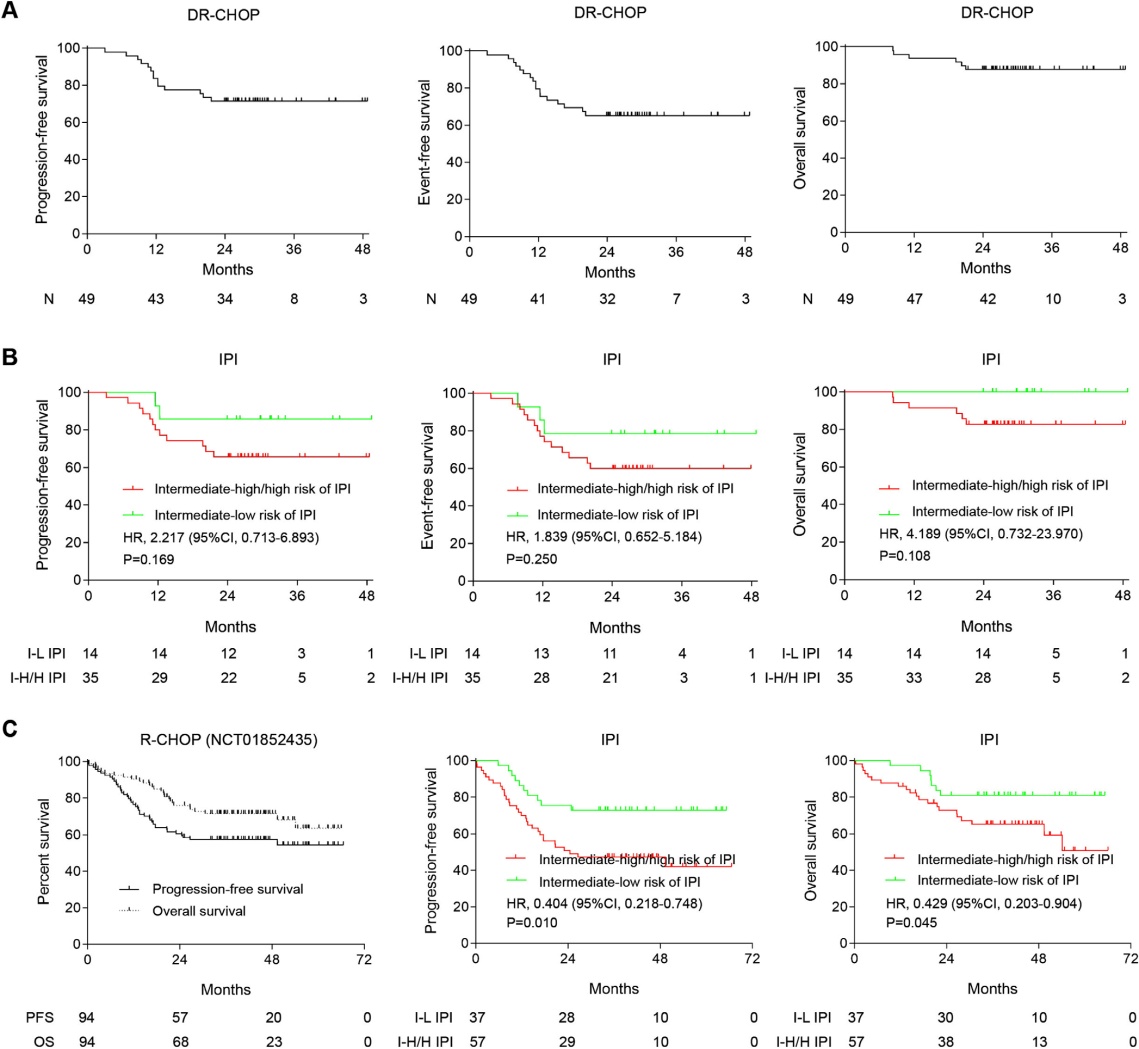
Outcomes of newly diagnosed diffuse large B cell lymphoma (DLBCL) patients received DR‐CHOP. (A) PFS, EFS and OS of all patients received DR‐CHOP. With a median follow‐up of 30.1 months (range 24.1–48.8), 2‐year PFS, EFS and OS rates were 71.4% (95% CI 56.6–82.0), 65.3% (95% CI 50.3–76.8) and 87.8% (95% CI 74.8–94.4), respectively. (B) PFS, EFS and OS stratified by international prognostic index. Two‐year PFS, EFS and OS rates were 65.7% (95% CI 47.6–78.9), 60.0% (95% CI 42.0–74.0) and 82.8% (95% CI 65.8–91.9) for those with intermediate‐high and high‐risk (international prognostic index [IPI] 3–5), comparable to 85.7% (95% CI 53.9–96.2), 78.6% (95% CI 47.2–91.5), and 100% for patients with intermediate‐low risk (IPI 2) (PFS, HR 2.217, 95% CI 0.713–6.893, *p* = 0.169; EFS, HR 1.839, 95% CI 0.652–5.184, *p* = 0.250; OS, HR 4.189, 95% CI 0.732–23.970, *p* = 0.108, respectively). (C) PFS and OS of IPI ≥ 2 patients received standard R‐CHOP (R‐CHOP50 and R‐CEOP70) in NCT01852435, as well as PFS and OS stratified by international prognostic index. DR‐CHOP = Decitabine, rituximab, cyclophosphamide, doxorubicin, vincristine and prednisone. PFS = Progression‐free survival. EFS = Event‐free survival. OS = Overall survival. HR = Hazard ratio. I‐L = Intermediate‐low risk. I‐H/H IPI = Intermediate‐high and high‐risk

To explore potential biomarkers related to clinical response, DNA‐sequencing and RNA‐sequencing were performed in patients with quality‐controlled tumour samples. Histone/DNA methylation gene mutations occurred in *KMT2D* (7/46, 15.2%), *KMT2C* (6/46, 13.0%), *TET2* (5/46, 10.9%), *HIST1H1C* (3/46, 6.5%) and *HIST1H1E* (3/46, 6.5%). Histone acetylation gene mutations occurred in *CREBBP* (3/46, 6.5%) and *EP300* (2/46, 4.3%). Chromatin remodeling gene mutations occurred in *ARID1A* (5/46, 10.9%) and *SGK1* (2/46, 4.3%). Interferon‐γ response pathway gene mutations occurred in *SOCS1* (7/46, 15.2%), *TP53* (5/46, 10.9%), *B2M* (4/46, 8.7%), *IRF8* (3/46, 6.5%) and *CIITA* (2/46, 4.3%). T‐cell activation gene mutations occurred in *PRDM1* (5/46, 10.9%), *TNFRSF14* (5/46, 10.9%), *BCL6* (4/46, 8.7%), *CD70* (3/46, 6.5%) and *MPEG1* (2/46, 4.3%). BCR/NF‐κB pathway gene mutations occurred in *TNFAIP3* (9/46, 19.6%), *DTX1* (7/46, 15.2%), *MYD88* (7/46, 15.2%), *CARD11* (3/46, 6.5%), *CD79B* (2/46, 4.3%), *PTPN6* (2/46, 4.3%) and *ZNF608* (2/46, 4.3%). WNT pathway gene mutations occurred in *PIM1* (8/46, 17.4%), *DDX3X* (3/46, 6.5%), *GNA13* (3/46, 6.5%) and *TBL1XR1* (2/46, 4.3%). JAK‐STAT pathway gene mutations occurred in *STAT6* (4/46, 8.7%) and *STAT3* (2/46, 4.3%). PI3K‐AKT pathway gene mutations occurred in *ATM* (3/46, 6.5%), *TSC2* (3/46, 6.5%) and *MTOR* (2/46, 4.3%). Cell cycle pathway gene mutations occurred in *CCND3* (5/46, 10.9%), *BTG1* (5/46, 10.9%), *BTG2* (4/46, 8.7%), *EBF1* (3/46, 6.5%), *FAS* (2/46, 4.3%) and *NFKBIE* (2/46, 4.3%) (Figure 2A). Univariate hazard estimates used unadjusted Cox proportional hazards models. Multivariate analysis included clinicopathological parameters and gene mutations demonstrating significance with *p* < 0.05 on univariate analysis. As expected, adverse prognostic effect of histone/DNA methylation gene mutations was not observed. Interferon‐γ response pathway gene mutations were significantly related to prolonged PFS (*p* = 0.021) and EFS (*p* = 0.024) (Figure 2B) and independently predicted favorable PFS by multivariate analysis. Among interferon‐γ response genes, *SOCS1* mutations may induce interferon‐γ signaling and increase immune cell activation.^4^ IRF8 can modulate T‐helper cell differentiation and function.^5^ *B2M* and *CIITA* mutations impair human leukocyte antigen‐mediated cancer cell recognition and are responsible for cancer immune escape.^6^ Using RNA‐sequencing analysis, gene expression patterns of 14 patients with interferon‐γ response pathway gene mutations and 21 patients without mutation were further compared. As confirmed by gene ontology and gene set enrichment analysis (Figures 2C and S2A), multiple signaling pathways were upregulated in the mutation group, including T‐helper 1 type immune response, interferon‐γ production, response to interferon‐γ, T‐cell differentiation, T‐cell activation and response to tumour necrosis factor pathways. Similar signaling pathway signatures were also observed in 33 DR‐CHOP‐responding patients (28 complete remission and five partial remission), as compared to four non‐responding patients (two stable disease and two progressive disease, Figure S2B,C). This was consistent with previous report that low‐dose decitabine (10 mg/day for 5 days) increased circulating interferon‐γ‐expressing CD3^+^T cells in Hodgkin's lymphoma.^7^ Moreover, DNMTi may enhance interferon response in cancer through endogenous retroviruses.^8^ These findings indicated that the microenvironment influence on interferon‐γ response and T‐cell activation were closely related to clinical response of DR‐CHOP. Functionally, interferon‐γ sensitivity of lymphoma cells is enhanced by interferon‐γ receptor 2, which is fundamental for anti‐tumour response.^9^ Indeed, as shown in Figure 2D, patients with interferon‐γ response pathway gene mutations presented significantly increased interferon‐γ receptor 2 expression, relative to those without mutation (*p* = 0.018).

**FIGURE 2:**
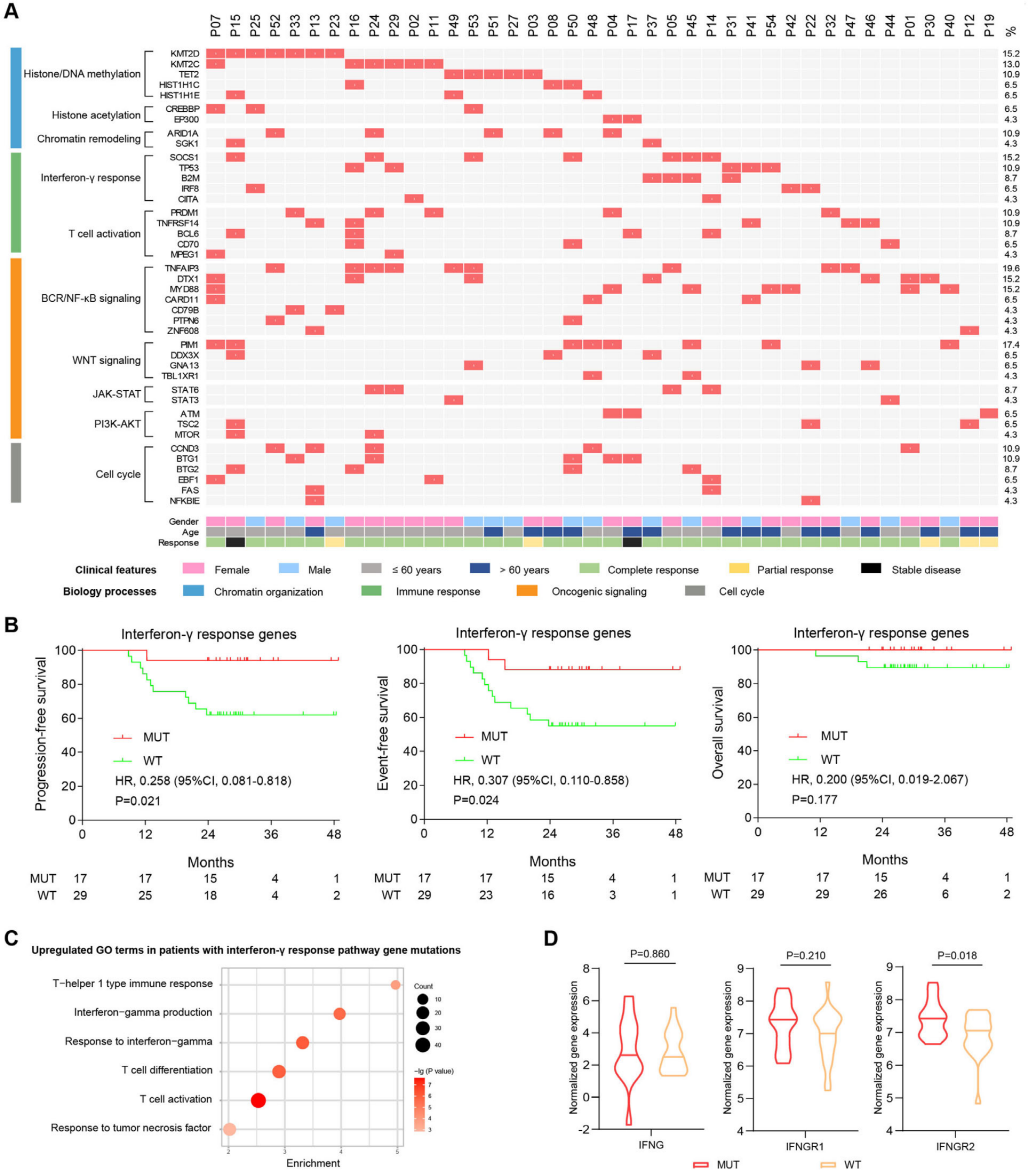
Correlation of gene mutation pattern with outcome of diffuse large B cell lymphoma (DLBCL) patients received DR‐CHOP. (A) Gene mutations identified by whole exome sequencing (*n* = 20), whole genome sequencing (*n* = 2) and targeted sequencing (*n* = 24) in 46 patients. Only genes mutated in two or more patients were shown. The percentage of gene mutations was listed on the right. (B) PFS, EFS and OS of patients with or without interferon‐γ response gene mutations. Two‐year PFS, EFS and OS rates were 94.1% (95% CI 65.0–99.1), 88.2% (95% CI 60.1–96.9) and 100% for patients with interferon‐γ response gene mutations, and 62.1% (95% CI 42.1–76.9), 55.2% (95% CI 35.6–71.0) and 89.6% (95% CI 71.3–96.5) for those without mutations (PFS: HR 0.258, 95% CI 0.081–0.818, *p* = 0.021; EFS: HR 0.307, 95% CI 0.110–0.858, *p* = 0.024; OS: HR 0.200, 95% CI 0.019–2.067, *p* = 0.177). (C) Upregulated gene ontology terms in patients with interferon‐γ response gene mutations relative to those without mutation. (D) Normalized gene expression of interferon‐γ (IFNG), interferon‐γ receptor 1 (IFNGR1) and interferon‐γ receptor 2 (IFNGR2) in tumour samples of patients with interferon‐γ genes mutation relative to those without mutation. DR‐CHOP = Decitabine, rituximab, cyclophosphamide, doxorubicin, vincristine and prednisone. PFS = Progression‐free survival. EFS = Event‐free survival. OS = Overall survival. HR = Hazard ratio. BCR/NF‐κB = B cell receptor / nuclear factor kappa B. JAK‐STAT = Janus kinase / signal transducers and activators of transcription. PI3K‐AKT = Phosphatidylinositol 3 kinase ‐ protein kinase B. MUT = Mutant. WT = Wild‐type

TP53 is critically involved in tumour progression, including DLBCL. Decitabine shows promising efficacy in treating patients with acute myeloid leukemia or myelodysplastic syndromes through targeting *TP53* mutations.^10^ It is worth notifying that all five DLBCL patients with *TP53* mutation achieved complete response and remained progression‐free till last follow‐up (Figure 3A). The possible structure‐function relationship of TP53 was addressed using the crystal structure of the protein. TP53 K132R, F134C, R175H, G187fs, F212fs, R282W and E285K could disrupt DNA binding domain (Figure 3B). Moreover, significant elevation of peripheral CD3^+^T, CD3^+^CD4^+^T, CD3^+^CD8^+^T cells and serum interferon‐γ were observed in mutant (MUT)‐TP53 patients, as other DR‐CHOP‐responding patients (Figure 3C). To further determine the microenvironment influence of decitabine on MUT‐TP53 DLBCL, SU‐DHL‐4*^TP53‐R248Q^*, SU‐DHL‐4*^TP53‐R273C^*, SU‐DHL‐4*^TP53‐R175H^* and wild‐type (WT)‐TP53 SU‐DHL‐4*^TP53‐WT^* cells were established. Upon treatment with decitabine (330 nM) for 5 days and doxorubicin (key cytotoxic agent of R‐CHOP, 200 nM) for 2 days at clinically achievable concentrations,^11^ T‐helper 1 cells were significantly increased in the co‐culture system of MUT‐TP53 cells (SU‐DHL‐4*^TP53‐R248Q^*, SU‐DHL‐4*^TP53‐R273C^* and SU‐DHL‐4*^TP53‐R175H^*) with peripheral blood mononuclear cells (*p* < 0.001, Figure 3D), which was not observed in SU‐DHL‐4*^TP53‐WT^* cells (*p* = 0.057, Figure 3E). As mechanism of action, T‐helper 1 cells secrete interferon‐γ and exhibit anti‐tumour activities during cell‐mediated adaptive immune response.^9^ Accordingly, significantly increased interferon‐γ production was observed in all MUT‐TP53 cells (*p* < 0.001), but not in WT‐TP53 cells (*p* = 0.105) upon treatment with decitabine and doxorubicin (Figure 3D,E). Therefore, decitabine could modulate the tumour microenvironment of *TP53‐*mutated DLBCL through enhancing T‐helper 1‐mediated anti‐tumour response.

**FIGURE 3:**
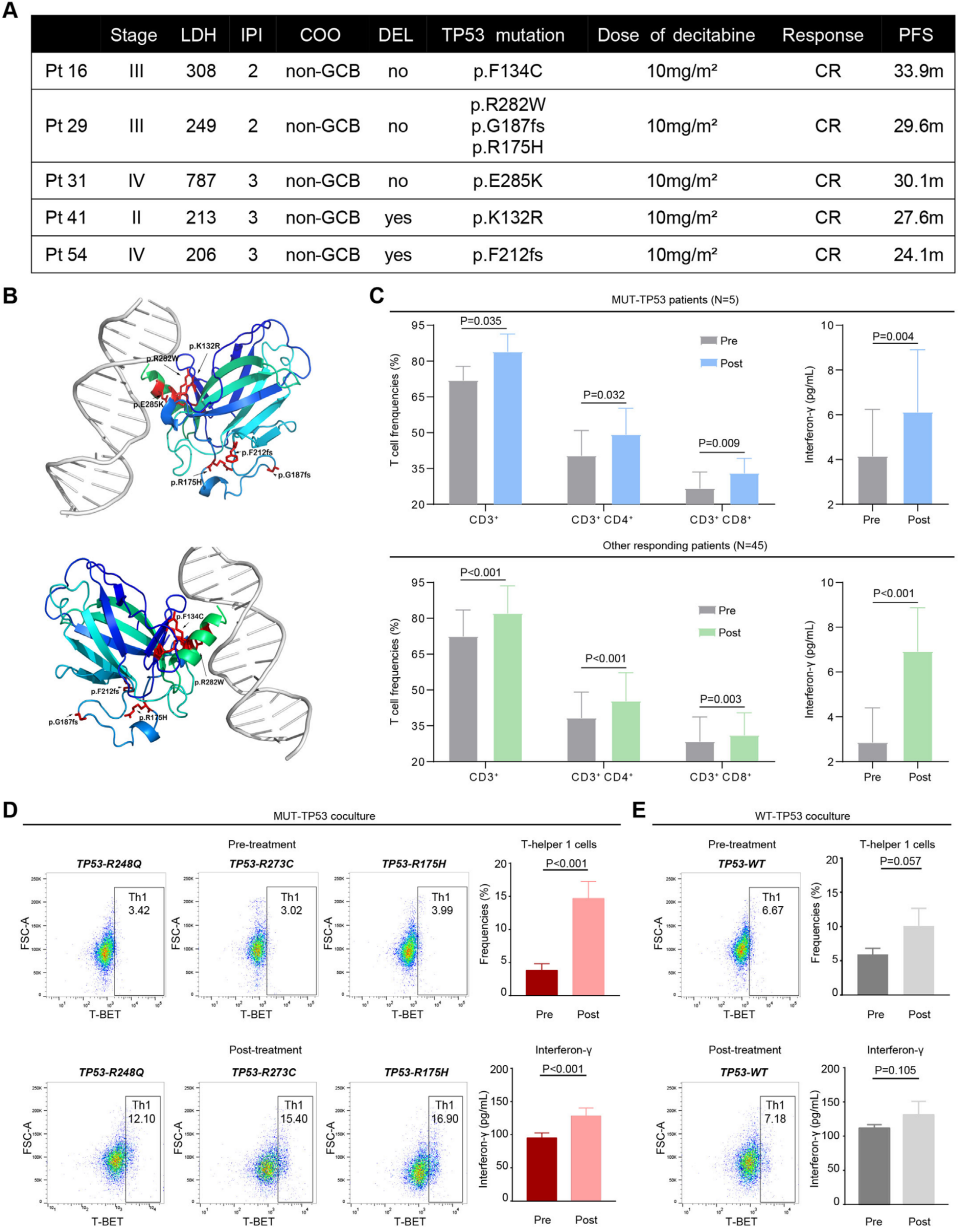
Influence of decitabine on *TP53* mutation and tumour microenvironment in diffuse large B cell lymphoma (DLBCL). (A) Basic characteristic of five patients with MUT‐TP53 in this study. (B) Structure prediction of the missense *TP53* mutations in this study. (C) Peripheral CD3^+^T cells pre‐ and post‐decitabine treatment in the first cycle by flow cytometry (left); as well as serum levels of interferon‐γ pre‐ and post‐ decitabine treatment in the first cycle (right) of MUT‐TP53 patients and other DR‐CHOP‐responding patients. (D) Comparison of T‐helper 1 cell percentage and interferon‐γ level in the co‐culture system of MUT‐TP53 (SU‐DHL‐4*^TP53‐R248Q^*, SU‐DHL‐4*^TP53‐R273C^*, SU‐DHL‐4*^TP53‐R175H^*) cells with peripheral blood mononuclear cells pre‐ and post‐treatment with decitabine and doxorubicin. (E) Comparison of T‐helper 1 cell percentage and interferon‐γ level in the co‐culture system of WT‐TP53 (SU‐DHL‐4*^TP53‐WT^*) cells with peripheral blood mononuclear cells pre‐ and post‐treatment with decitabine and doxorubicin. DR‐CHOP = Decitabine, rituximab, cyclophosphamide, doxorubicin, vincristine and prednisone. MUT‐TP53 = Mutant TP53. WT‐TP53 = Wild‐type TP53

In conclusion, DR‐CHOP was effective and safe in newly diagnosed DLBCL patients. Benefit impact of DR‐CHOP on the tumour microenvironment further provided clinical rationale of targeting DNA methylation as an important immunomodulatory strategy in treating DLBCL.

## CONFLICT OF INTEREST

The authors declare that they have no competing interests.

## FUNDING INFORMATION

National Natural Science Foundation of China, Grant Numbers: 82130004, 81830007, 82170178, 81670176 and 82070204; Chang Jiang Scholars Program; Shanghai Municipal Education Commission Gaofeng Clinical Medicine Grant Support, Grant Numbers: 20152206 and 20152208; Clinical Research Plan of Shanghai Hospital Development Center, Grant Number: SHDC2020CR1032B; Multicenter Clinical Research Project by Shanghai Jiao Tong University School of Medicine, Grant Number: DLY201601; Collaborative Innovation Center of Systems Biomedicine; Samuel Waxman Cancer Research Foundation

## Supporting information

Supporting information

## References

[1] Miao Y , Medeiros LJ , Li Y , Li J , Young KH . Genetic alterations and their clinical implications in DLBCL. Nat Rev Clin Oncol. 2019;16(10):634‐652. 31127191 10.1038/s41571-019-0225-1

[2] Xu PP , Fu D , Li JY , et al. Anthracycline dose optimisation in patients with diffuse large B‐cell lymphoma: a multicentre, phase 3, randomised, controlled trial. Lancet Haematol. 2019;6(6):e328‐e337. 31126528 10.1016/S2352-3026(19)30051-1

[3] Lue JK , O'Connor OA . A perspective on improving the R‐CHOP regimen: from mega‐CHOP to ROBUST R‐CHOP, the PHOENIX is yet to rise. Lancet Haematol. 2020;7(11):e838‐e850. 33091357 10.1016/S2352-3026(20)30222-2

[4] Lee PY , Platt CD , Weeks S , et al. Immune dysregulation and multisystem inflammatory syndrome in children (MIS‐C) in individuals with haploinsufficiency of SOCS1. J Allergy Clin Immunol. 2020;146(5):1194‐1200. 32853638 10.1016/j.jaci.2020.07.033 PMC7445138

[5] Ouyang X , Zhang R , Yang J , et al. Transcription factor IRF8 directs a silencing programme for TH17 cell differentiation. Nat Commun. 2011;2:314. 21587231 10.1038/ncomms1311 PMC3112536

[6] Pizzi M , Boi M , Bertoni F , Inghirami G . Emerging therapies provide new opportunities to reshape the multifaceted interactions between the immune system and lymphoma cells. Leukemia. 2016;30(9):1805‐1815. 27389058 10.1038/leu.2016.161

[7] Li X , Zhang Y , Chen M , et al. Increased IFNγ(+) T cells are responsible for the clinical responses of low‐dose DNA‐demethylating agent decitabine antitumor therapy. Clin Cancer Res. 2017;23(20):6031‐6043. 28706011 10.1158/1078-0432.CCR-17-1201

[8] Chiappinelli KB , Strissel PL , Desrichard A , et al. Inhibiting DNA methylation causes an interferon response in cancer via dsRNA including endogenous retroviruses. Cell. 2015;162(5):974‐986. 26317466 10.1016/j.cell.2015.07.011 PMC4556003

[9] Ivashkiv LB . IFNγ: signalling, epigenetics and roles in immunity, metabolism, disease and cancer immunotherapy. Nat Rev Immunol. 2018;18(9):545‐558. 29921905 10.1038/s41577-018-0029-z PMC6340644

[10] Welch JS , Petti AA , Miller CA , et al. TP53 and decitabine in acute myeloid leukemia and myelodysplastic syndromes. N Engl J Med. 2016;375(21):2023‐2036. 27959731 10.1056/NEJMoa1605949 PMC5217532

[11] Clozel T , Yang S , Elstrom RL , et al. Mechanism‐based epigenetic chemosensitization therapy of diffuse large B‐cell lymphoma. Cancer Discov. 2013;3(9):1002‐1019. 23955273 10.1158/2159-8290.CD-13-0117 PMC3770813

