# Supplementary material for: Clinical efficacy and tumour microenvironment influence of decitabine plus R‐CHOP in patients with newly diagnosed diffuse large B‐Cell lymphoma: Phase 1/2 and biomarker study
Source: Clin Transl Med. 2021 Dec 19;11(12):e584. doi: 10.1002/ctm2.584 (PMC8684715; doi:10.1002/ctm2.584)
Supplement: Supplementary file 1 — Supporting information [file CTM2-11-e584-s001.docx]

**Supplemental Materials**

**Clinical Efficacy and Tumor Microenvironment Influence of Decitabine Plus R-CHOP in Patients with Newly Diagnosed Diffuse Large B-Cell Lymphoma: Phase 1/2 and Biomarker Study**

Mu-Chen Zhang^1*^, Ying Fang^1*^, Peng-Peng Xu^1*^, Lei Dong^2*^, Rong Shen^1^, Yao-Hui Huang^1^, Di Fu^1^, Zi-Xun Yan^1^, Shu Cheng^1^, Xu-Feng Jiang^3^, Qi Song^4^, Yang He^1^, Yan Zhao^1^, Min Lu^1^, Jing Ye^1^, Feng Liu^1^, Lin Cheng^1^, Chao-Fu Wang^2^, Li Wang^1,5#^, and Wei-Li Zhao^1,5#^

^*^ These authors contributed equally to this manuscript.

^1^ Shanghai Institute of Hematology, State Key Laboratory of Medical Genomics, National Research Center for Translational Medicine at Shanghai, Ruijin Hospital affiliated to Shanghai Jiao Tong University School of Medicine, Shanghai, China

^2^ Department of Pathology, Ruijin Hospital affiliated to Shanghai Jiao Tong University School of Medicine, Shanghai, China

^3^ Department of Nuclear Medicine, Ruijin Hospital affiliated to Shanghai Jiao Tong University School of Medicine, Shanghai, China

^4^ Department of Radiology, Ruijin Hospital affiliated to Shanghai Jiao Tong University School of Medicine, Shanghai, China

^5^ Pôle de Recherches Sino-Français en Science du Vivant et Génomique, Laboratory of Molecular Pathology, Shanghai, China

^#^ Correspondence to: Wei-Li Zhao, Email: zhao.weili@yahoo.com, Li Wang, Email: wl_wangdong@126.com.

Shanghai Institute of Hematology, State Key Laboratory of Medical Genomics, National Research Center for Translational Medicine at Shanghai, Ruijin Hospital affiliated to Shanghai Jiao Tong University School of Medicine, Shanghai, China

**
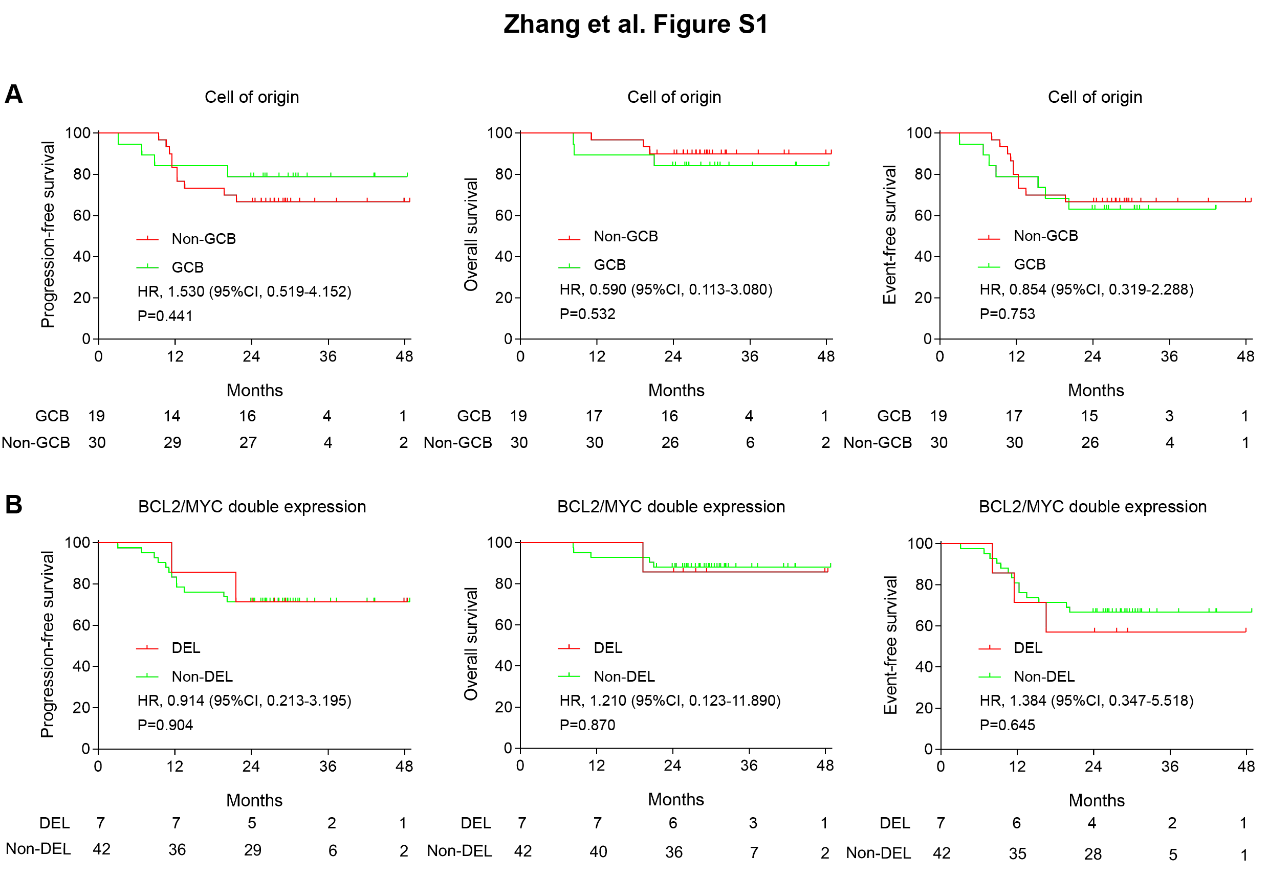
**

**Figure S1. Outcomes of patients received DR-CHOP based on different subgroups.**

(A) Progression-free survival, overall survival, and event-free survival stratified by cell of origin.

(B) Progression-free survival, overall survival, and event-free survival stratified by BCL2/MYC double expression.

DR-CHOP=Decitabine, rituximab, cyclophosphamide, doxorubicin, vincristine and prednisone. GCB=Germinal center B-cell. DEL=Double expression lymphoma.

**
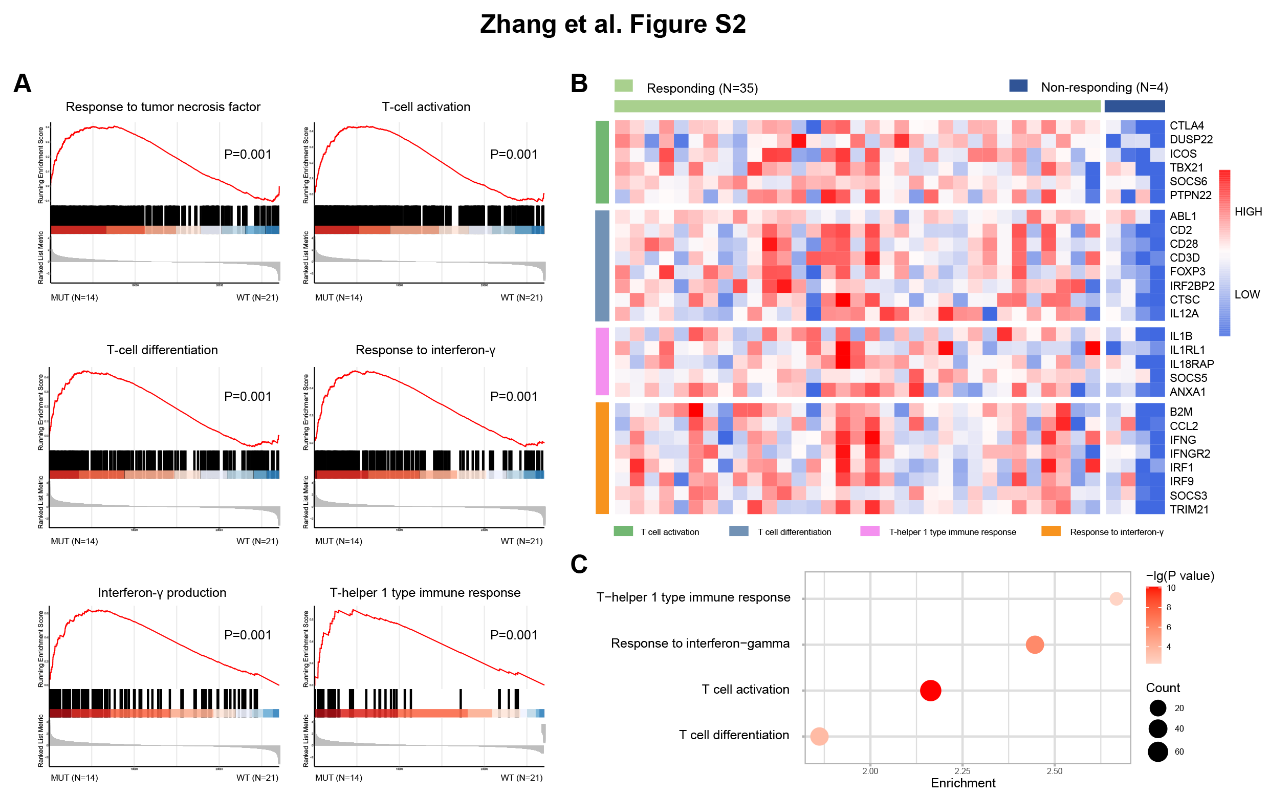
**

**Figure S2. Gene expression signatures and peripheral blood T-cell activity related to response to DR-CHOP.**

(A) Gene set enrichment analysis of patients with interferon-γ response gene mutations relative to those without mutation.

(B) Profile of signaling pathways according to response to DR-CHOP.

(C) Upregulated Gene Ontology terms in responding patients relative to non-responding patients.

DR-CHOP=Decitabine, rituximab, cyclophosphamide, doxorubicin, vincristine and prednisone.

**Table S1. Adverse events of the enrolled patients**

|  | **Phase 1*** | | **Phase 2**** | | |
| --- | --- | --- | --- | --- | --- |
|  | **Grade 1-2** | **Grade 3-4** | | **Grade 1-2** | **Grade 3-4** |
| **Number of patients with hematological adverse events** | | | | |  |
| Neutropenia | 1 (9.1%) | 10 (90.9%) | | 12 (24.5%) | 37 (75.5%) |
| Anemia | 3 (27.3%) | 6 (54.5%) | | 18 (36.7%) | 5 (10.2%) |
| Thrombocytopenia | 4 (36.4%) | 4 (36.4%) | | 22 (44.9%) | 6 (12.2%) |
| Febrile neutropenia | 1 (9.1%) | 1 (9.1%) | | 2 (4.1%) | 5 (10.2%) |
| **Number of patients with non-hematological adverse events** | | | | | |
| Elevated ALT/AST | 5 (45.5%) | 1 (9.1%) DLT | | 12 (24.5%) | 4 (8.2%) |
| Elevated AKP/GGT | 2 (18.2%) | 0 | | 8 (16.3%) | 0 |
| Nausea | 2 (18.2%) | 0 | | 7 (14.2%) | 0 |
| Pulmonary infection | 1 (9.1%) | 0 | | 2 (4.1%) | 6 (12.2%) |
| Vomiting | 2 (18.2%) | 0 | | 5 (10.2%) | 0 |
| Fatigue | 1 (9.1%) | 0 | | 5 (10.2%) | 0 |
| Diarrhea | 1 (9.1%) | 0 | | 4 (8.2%) | 0 |
| Infusion-related reaction | 1 (9.1%) | 0 | | 4 (8.2%) | 0 |
| Constipation | 2 (18.2%) | 0 | | 2 (4.1%) | 0 |
| Neuropathy peripheral | 1 (9.1%) | 0 | | 3 (6.1%) | 0 |
| C. albicans bloodstream infection | 0 | 1 (9.1%) DLT | | 0 | 0 |

* Adverse events in phase 1 patients (n=11) after 66 cycles of DR-CHOP.

** Adverse events in evaluable patients (n=49) after 280 cycles of DR-CHOP (including 6 patients from phase 1 who were treated with 10 mg/m² decitabine).

Data are number of patients (%). All grade 1-4 adverse events are shown. DLT=dose-limiting toxicity.

**Supplemental Results**

**Patient characteristics and toxicity in phase 1 portion**

From December 2016 to July 2017, 11 patients were enrolled in the phase 1 portion of the study, 72.7% of whom were female, 90.9% with advanced Ann Arbor stage, 81.8% showed elevated serum lactate dehydrogenase (LDH) level, and 81.8% had multiple extranodal involvement (Table 1). The median age was 46 years (range 25-57). According to the 3+3 escalation design, patients were enrolled to the cohort 1 and 2 as follows: in the cohort 1 of 10mg/m^2^, none of the first three patients experienced DLT. In the cohort 2 of 15mg/m^2^, one of the first three patients experienced a DLT (grade 3 increased ALT and AST), leading to the enrollment of additional two patients of 15mg/m^2^ (one of these two patients experienced another DLT as bloodstream infection of C. albicans). Thus, no additional patient was enrolled at 15mg/m², and three more patients were added to cohort 1 of 10mg/m^2^. None of the six patients in cohort 1 experienced DLT and the MTD of decitabine was identified as 10mg/m².

Patients in phase 1 received a total of 66 treatment cycles of DR-CHOP. Hematological and non-hematological AEs were summarized in Table S1. For hematological toxicities, grade 3-4 neutropenia was present in 90.9% of the patients, grade 3 anemia was observed in 54.5% of the patients, and grade 3 thrombocytopenia was observed in 36.4% of the patients, without bleeding complications. No grade 4 anemia or thrombocytopenia was reported in phase 1 portion. Febrile neutropenia occurred in 18.2% of the patients (two events) and was of grade 3 (9.1%, one event) in maximum. Grade 3 non-hematological toxicities included elevated ALT/AST (one event, DLT) and bloodstream infection of C. albicans (one event, DLT). No grade 4 non-hematological toxicity was reported. The two patients who experienced DLT received 10 mg/m² decitabine from the second cycle of chemotherapy. Since the MTD of decitabine was established, all of the other three patients of cohort 2 received 10mg/m² decitabine in the following cycles of DR-CHOP.

**Patient characteristics and toxicity in phase 2 portion**

Six patients from phase 1 portion who received 10mg/m² decitabine (the established MTD) were counted as evaluable patients. For phase 2, an additional 43 patients were enrolled from August 2017 to November 2018, for a total of 49 evaluable patients treated at the MTD (Table 1). Among 49 evaluable patients, the median age was 55 years (range 25-74) and 27 patients (55.1%) were female. Forty-four patients (89.8%) presented advanced Ann Arbor stage and 43 patients (87.8%) showed elevated serum LDH level. Thirty-seven patients (75.5%) had multiple extranodal involvement, mainly as bone marrow (24.5%), gastrointestinal (16.3%), and bone (16.3%). Thirty-five patients (71.4%) were of intermediate-high or high-risk of IPI at diagnosis. Thirty patients (61.2%) were classified as non-germinal center B-cell (non-GCB) subtype and seven patients (14.3%) had BCL2/MYC double expression without MYC or BCL2/BCL6 translocation. One patient had MYC/BCL2/BCL6 triple-hit lymphoma. No significant difference of clinical and pathological parameter was observed among patients of phase 1 portion, phase 2 portion, or evaluable cohort.

Of the 294 planned treatment cycles of DR-CHOP, 280 (95.2%) were given to the 49 evaluable patients. Hematological and non-hematological AEs were summarized in Table S1. For hematological AEs, grade 3 and 4 neutropenia were present in 18.4% and 57.1% of the patients, respectively. G-CSF prophylaxis (pegfilgrastim 6 mg subcutaneously) was given from the second cycle of DR-CHOP if grade ≥ 3 neutropenia was present in the first cycle. Grade 3 and 4 neutropenia occurring in 2 or more cycles were observed 28.6% of patients, with a median duration of 3 days (range, 3-6). Grade 3 anemia was observed in 10.2% of the patients. Grade 3 and 4 thrombocytopenia were observed in 8.1% and 4.1% of the patients, respectively, without bleeding complications. Grade 3 febrile neutropenia occurred in 10.2% of the patients. The most common grade 1-2 non-hematological AEs were ALT/AST (24.5%) and AKP/gamma glutamyl transpeptidase (GGT) elevation (16.3%). Grade 3 ALT/AST elevation was observed in 8.2% of the patients. Grade 3 pulmonary infection was observed in 12.2% of the patients. No grade 4 non-hematological toxicities was reported.

Nine of the 49 evaluable patients (18.4%) needed cyclophosphamide and doxorubicin dose reduction, because of treatment delay due to grade 3 infection (6 patients, 12.2%) and grade 4 neutropenia (3 patients, 6.2%). No death occurred during the study as a result of toxicity related to treatment. Overall, six patients died of lymphoma progression.

**Outcomes of patients based on different subgroups**

Fourteen of 19 (73.7%) patients with GCB subtype and 25 of 30 (83.3%) patients with non-GCB subtype achieved complete response. Two-year PFS, OS, and EFS rates were 78.9% (95%CI 53.2-91.5), 84.2% (95%CI 58.6-94.6), 63.2% (95%CI 37.9-80.4) in the GCB group, and 66.7% (95%CI 46.9-80.5), 90.0% (95%CI 72.1-96.7), 66.7% (95%CI 46.9-80.5) in the non-GCB group (PFS, HR 1.530, 95%CI 0.519-4.152, P=0.441; OS, HR 0.590, 95%CI 0.113-3.080, P=0.532; EFS, HR 0.854, 95%CI 0.319-2.288, P=0.753, respectively, Figure S1A).

Five of 7 (71.4%) DEL patients and 34 of 42 (81.0%) non-DEL patients achieved complete response. Two-year PFS, OS, and EFS rates were 71.4% (95%CI 25.8-92.0), 85.7% (95%CI 33.4-97.8), 57.1% (95%CI 17.2-83.7) in the DEL group, and 71.4% (95%CI 55.2-82.6), 88.1% (95%CI 73.7-94.9), 66.7% (95%CI 50.3-78.7) in the non-DEL group (PFS, HR 0.914, 95%CI 0.213-3.915, P=0.904; OS, HR 1.210, 95%CI 0.123-11.890, P=0.870; EFS, HR 1.384, 95%CI 0.347-5.518, P=0.645, respectively, Figure S1B).

**Supplemental Methods**

**Study design and participants**

This was an investigator-initiated, open-label, single-arm, phase 1/2 trial (NCT02951728). Patients were eligible if they were 15-75 years; had newly diagnosed, histologically confirmed CD20-positive DLBCL, an Eastern Cooperative Oncology Group (ECOG) performance status of 0-2, IPI risk of intermediate or high (IPI ≥ 2), and a life expectancy of more than 6 months. Patients were excluded if they had previous chemotherapy or stem-cell transplantation; history of malignancy (other than skin cancers or carcinomas in situ of the cervix); uncontrollable cardio-cerebral vascular, coagulation, autoimmune, infectious disease; primary central nervous system (CNS) lymphoma; left ventricular ejection fraction ≤ 50% ^[1,2]^. They were also eliminated from the study if, at enrollment, their neutrophil count < 1.5 × 10^9^/L, platelets < 75 × 10^9^/L, alanine aminotransferase (ALT) or aspartate aminotransferase (AST) > 2 × upper limit of normal (ULN), alkaline phosphatase (AKP) or bilirubin > 1.5 × ULN, or creatinine > 1.5 × ULN (unless they were caused by the lymphoma). They were not enrolled if they were unable to comply with the protocol for mental or other unknown reasons; pregnancy or lactation; or positive for hepatitis B virus (HBV-DNA) and human immunodeficiency virus.

Pathological diagnosis was performed according to the 2016 World Health Organization classification ^[3]^. Cell of origin profile was determined by Hans algorithm, with 30% cut-off value of CD10, BCL6, and MUM-1 ^[4]^. As for BCL2/MYC double expression lymphoma (DEL), cut-off value of BCL2 and MYC were 50% and 40%, respectively ^[3]^. Fluorescence in-situ hybridization of BCL2, BCL6, and MYC rearrangements were performed for each patient. The peripheral blood was collected before (Day 0) and after decitabine treatment (Day 6) during the first cycle. In the exploratory correlative biomarker analyses, all enrolled patients with qualified tumor samples were included. An association between disease outcome (complete response, partial response, stable disease, and progression disease) with gene mutation status, signaling pathway enrichment, and tumor microenvironment was assessed.

**Procedures**

In phase 1 portion of the study, the standard 3+3 dose-escalation design was applied. Patients were assigned to increasing doses of decitabine (10mg/m², 15mg/m², and 20mg/m²) given intravenously on days 1-5, prior to R-CHOP (rituximab 375 mg/m² intravenously on day 6, cyclophosphamide 750mg/m², doxorubicin 50mg/m², and vincristine 1.4mg/m² [maximum 2.0mg] intravenously on day 7, prednisone 60mg/m² [maximum 100mg] orally on days 7-11 of every 28-day cycle). Decitabine dose escalation was under the standard 3+3 design, with at least 3 patients enrolled into each cohort. A 28-day (i.e., 1-cycle) observation period was allowed for evaluation of dose-limiting toxicities (DLTs). If none of the first 3 evaluable patients experienced a DLT, the next dose cohort was opened. If a DLT was observed in 1 patient, additional patients were enrolled at that dose level until at least 6 evaluable patients had completed the DLT observation window or a second DLT occurred. If no additional DLT was reported, the next dose could be evaluated. If an additional DLT was observed after cohort expansion, further enrollment on that dosing schedule was halted and that dose was either declared as exceeding the maximum tolerated dose (MTD). In phase 2 portion of the study, all patients were assigned to receive decitabine at the MTD in combination with R-CHOP.

After cycle 1, we allowed a dose delay up to 7 days. Decitabine dose reduction would be undertaken in case of grade 3 non-hematologic toxicity or grade 4 hematologic toxicity (including neutropenia lasting 7 days if patients were receiving granulocyte-colony stimulating factor [G-CSF]). At the first qualifying toxicity, the dose would be reduced from the MTD to the next lower dose, with subsequent reductions of one dose level for each additional qualifying toxicity. A patient who experienced such toxicity at the lowest dose level will be removed from the study and would continue treatment as per the treating physician. For dose adjustments with R-CHOP components, the investigator referred to the prescribing information for the respective chemotherapeutic agent.

G-CSF prophylaxis (pegfilgrastim 6 mg subcutaneously) was given from the second cycle of chemotherapy if grade ≥ 3 neutropenia was present in the first cycle. Lamivudine was administered in occult carriers of HBV to prevent HBV reactivation. Prophylaxis for CNS relapse was given to patients with involvement of bone marrow, nasal or paranasal sinuses, orbit, breast, or testis. Tumor lysis prophylaxis and radiation therapy were performed for patients with bulky disease, or with residual disease at the end of treatment, at the discretion of physicians.

**Outcomes**

The primary endpoint for phase 1 was to determine the MTD of decitabine when given in combination with R-CHOP, defined as the highest dose resulting in DLTs in less than one-third of patients in a cohort of 6 or more patients. The MTD for decitabine when combined with R-CHOP were to be taken forward as the recommended phase 2 dose for the respective arm, unless there was any safety or tolerability indication that a lower dose would be more appropriate. The primary endpoint for phase 2 was complete response rate assessed by PET-CT. Secondary endpoints were overall response rate, PFS, OS, EFS, safety and tolerability.

Patients were monitored weekly for adverse events (AEs), which were graded for severity with the National Cancer Institute Common Terminology Criteria for Adverse Events, Version 4.0 (NCI CTCAE v4.0). DLT was defined as the occurrence of any of the following: any grade 3 or greater non-hematological toxicity at least possibly related to study drug, grade 4 anemia, thrombocytopenia or neutropenia for more than 7 days within cycle 1. The following AEs were excluded as DLTs: grade 3/4 neutropenia not accompanied by fever and improved to grade 2 within 7 days, grade 3 febrile neutropenia.

Treatment response was assessed according to a modified version of Lugano 2014 criteria ^[5]^. Response assessment by PET-CT occurred after cycle 3 and at end of treatment. CT of the neck, thorax, abdomen, and pelvis was repeated every 3 months thereafter to monitor disease progression until 1 year, then every 6 months until 2 years, and every year thereafter. PFS was measured from diagnosis to date of progression, relapse, or death from any cause. OS was measured from diagnosis to death of any cause or date of last follow-up. EFS was measured from diagnosis to date of progression, relapse, death from any cause, initiation of new treatment without progression, or treatment discontinuation ^[6]^. Patients with a partial response who were given an additional treatment (i.e., radiotherapy) without apparent disease progression were not considered as an event for PFS analysis.

**Historical control cohort**

Patients from 20 centers of the Multicenter Hematology/Oncology Programs Evaluation System (M-HOPES) in China with newly diagnosed DLBCL**,** treated with regular R-CHOP50 (doxorubicin 50mg/m^2^) or regular R-CEOP70 (rituximab, cyclophosphamide, epirubicin 70mg/m^2^, vincristine, and prednisone) between May 15, 2013 and March 16, 2016 in our previous multicenter, phase III, randomized, controlled trial (NCT01852435)^[7]^, and who met the same inclusion/exclusion criteria as those treated with DR-CHOP were referred as the historical control cohort and analyzed for outcome on the basis of DLBCL subtypes.

**DNA-sequencing**

Whole exome sequencing (WES) (n=20) and whole genome sequencing (WGS) (n=2) were performed on frozen tumor samples of 22 patients with genomic DNA extracted using Wizard® Genomic DNA Purification Kit (Promega, Wisconsin-Madison, USA). Targeted sequencing was performed on formalin-fixed paraffin-embedded tumor samples of 24 patients with genomic DNA extracted using GeneRead DNA formalin-fixed paraffin-embedded Tissue Kit (Qiagen, Hilden, Germany). A targeted sequencing panel was assessed using a custom SureSelect library (Agilent Technologies), including 55 genes related to pathogenesis of DLBCL according to literature, or associated with significant alternations in gene expression revealed by RNA-sequencing.

**RNA-sequencing**

RNA-sequencing was performed on frozen tumor samples of 37 patients with RNA extracted using RNeasy MinElute Cleanup Kit (Qiagen, Dusseldorf, German). RNA integrity was assessed using RNA 6000 Nano Kit (Agilent, California, USA) on Agilent 2100 Bioanalyzer (Agilent, California, USA) and RNA-sequencing was performed using Illumina HiSeq 2000 (Illumina, California, USA). Bioinformatic analyses were performed by r 4.0.3, using R package “sva” to remove batch effect. Raw reads were normalized and differentially expressed genes (DEGs) were obtained with R package “limma” (v3.38.3).

**Gene set enrichment analysis (GSEA)**

GSEA results were presented as the upregulation or downregulation of the desired gene set using GSEA v4.1.0 software and Molecular Signature Database (MSigDB) ^[8,9]^. The metric method used to rank the genes was Signal2Noise by default. Analysis was run on 1000 permutations to assess the statistical significance of the enrichment score, as recommended by the GSEA team (<http://www.broadinstitute.org/gsea>). Pathways were considered statistically significant when the P value was < 0.05 and the false discovery rate was < 0.25.

**Interferon-γ measurement by ELISA**

Serum specimens were collected before (day 0) and after decitabine treatment (day 6) during the first cycle. Concentration of interferon-γ was measured in serum using an interferon-γ ELISA kit (cat. no. 70-EK180-48, MultiSciences Biotechnology) according to the manufacturer's protocol. Cytokine concentration was determined by measuring optical density using a microplate reader at 450/570 nm.

***In vitro*** **co‐culture system**

B‐lymphoma cell line SU-DHL-4 was obtained from American Type Culture Collection (Manassas, VA, USA). Peripheral blood mononuclear cells were isolated from peripheral blood, as previously described ^[10]^. Cell lines were grown in RPMI-1640 medium supplemented with 10% heat-inactivated fetal bovine serum and 1% penicillin/streptomycin (15140122, Gibco, Carlsbad, CA, USA) in a humidified atmosphere containing 95% air-5% CO_2_ at 37 °C.

**Cell transfection**

*TP53* gene in SU-DHL-4 cell line was deleted by CRISPR/Cas9 system. SU-DHL-4 cells were then transfected with viral particles containing purified plasmids expressing GV392-U6-sgRNA-EF1a-Cas9-FLAG-P2A-puro to generate SU-DHL-4*^TP53^*^-/-^. The stably transfected clones were selected by puromycin (10μg/uL). SU-DHL-4*^TP53^*^-/-^ cells were further transfected with viral particles containing purified plasmids pGV358-EGFR-puro-TP53-WT (SU-DHL-4*^TP53-WT^*), CV186-Cherry-puro-TP53-R248Q (SU-DHL-4*^TP53-R248Q^*), CV186-Cherry-puro-TP53-R273C (SU-DHL-4*^TP53-R273C^*), CV186-Cherry-puro-TP53-R175H (SU-DHL-4*^TP53-R175H^*), as well as vector controls CV186-Cherry-puro-TP53-vector (MOI=50) using Lipofectamine 3000 transfection reagents (Invitrogen, Shanghai, China), according to the manufacturer’s instruction. The stably transfected clones were selected by Cherry using BD FacsAria.

**Flow cytometry**

To detect percentage of immune cells, co‐cultured cells were stained with anti-CD4 (300554, BioLegend), anti-CD8 (560662, BD Pharmingen), anti-T-bet (644816, BioLegend) and anti-EOMES (IC6166A, R&D Systems). Data were analyzed using flowjo software (Becton Dickinson).

**Statistical analysis**

In phase 1, the MTD of decitabine when combined with R-CHOP was identified by a 3+3 algorithm. The expected enrollment for the dose-finding stage was 12-15 patients. In phase 2, the efficacy of the regimen was assessed by Simon’s two-stage minimax design ^[11]^. Our objective was to show a 15% improvement in frequency of complete response with the new regimen relative to an expected frequency of 65% with R-CHOP alone ^[7]^, with an α of 0.05, 75% power. A total of 48 patients was required to obtain the hypothesis, 22 of whom were to be enrolled during stage 1 and 26 during stage 2. The study would be stopped early if 14 or fewer patients achieved complete response in stage 1. Similarly, if 36 or fewer patients achieved complete response by trial completion, the hypothesis would also be rejected.

Efficacy and safety analyses were by intention to treat. Statistical analyses were performed by Statistical Package for the Social Science (SPSS) 23.0 software (SPSS Inc., Chicago, IL, USA). Survival estimates were calculated by Kaplan-Meier method and survival curves were compared by Log-rank test. A two-sided P value of <0.05 was considered statistically significant.

**References**

[1]. Cunningham D, Hawkes EA, Jack A, et al. Rituximab plus cyclophosphamide, doxorubicin, vincristine, and prednisolone in patients with newly diagnosed diffuse large B-cell non-Hodgkin lymphoma: a phase 3 comparison of dose intensification with 14-day versus 21-day cycles. *Lancet.* 2013;381(9880):1817-1826.

[2]. Vitolo U, Trneny M, Belada D, et al. Obinutuzumab or Rituximab Plus Cyclophosphamide, Doxorubicin, Vincristine, and Prednisone in Previously Untreated Diffuse Large B-Cell Lymphoma. *J Clin Oncol.* 2017;35(31):3529-3537.

[3]. Swerdlow SH, Campo E, Pileri SA, et al. The 2016 revision of the World Health Organization classification of lymphoid neoplasms. *Blood.* 2016;127(20):2375-2390.

[4]. Hans CP, Weisenburger DD, Greiner TC, et al. Confirmation of the molecular classification of diffuse large B-cell lymphoma by immunohistochemistry using a tissue microarray. *Blood.* 2004;103(1):275-282.

[5]. Cheson BD, Fisher RI, Barrington SF, et al. Recommendations for initial evaluation, staging, and response assessment of Hodgkin and non-Hodgkin lymphoma: the Lugano classification. *J Clin Oncol.* 2014;32(27):3059-3068.

[6]. Cheson BD, Pfistner B, Juweid ME, et al. Revised response criteria for malignant lymphoma. *J Clin Oncol.* 2007;25(5):579-586.

[7]. Xu PP, Fu D, Li JY, et al. Anthracycline dose optimisation in patients with diffuse large B-cell lymphoma: a multicentre, phase 3, randomised, controlled trial. *Lancet Haematol.* 2019;6(6):e328-e337.

[8]. Subramanian A, Tamayo P, Mootha VK, et al. Gene set enrichment analysis: a knowledge-based approach for interpreting genome-wide expression profiles. *Proc Natl Acad Sci U S A.* 2005;102(43):15545-15550.

[9]. Vamsi K Mootha CML, Karl-Fredrik Eriksson, Aravind Subramanian, Smita Sihag, Joseph Lehar, Pere Puigserver, Emma Carlsson, Martin Ridderstråle, Esa Laurila, Nicholas Houstis, Mark J Daly, Nick Patterson, Jill P Mesirov, Todd R Golub, Pablo Tamayo, Bruce Spiegelman, Eric S Lander, Joel N Hirschhorn, David Altshuler, Leif C Groop. PGC-1alpha-responsive genes involved in oxidative phosphorylation are coordinately downregulated in human diabetes. *Nat Genet.* 2003;34(3):267-273.

[10]. Sun R, Zheng Z, Wang L, et al. A novel prognostic model based on four circulating miRNA in diffuse large B-cell lymphoma: implications for the roles of MDSC and Th17 cells in lymphoma progression. *Mol Oncol.* 2021;15(1):246-261.

[11]. Simon R. Optimal two-stage designs for phase II clinical trials. *Control Clin Trials.* 1989;10(1):1-10.
